# Efficacy of cilostazol in canine bradyarrhythmia

**DOI:** 10.3389/fvets.2022.954295

**Published:** 2022-08-16

**Authors:** Takahiro Ohmori, Yuri Matsumura, Aritada Yoshimura, Shohei Morita, Hiroshi Hasegawa, Daiki Hirao, Ryuji Fukushima

**Affiliations:** Animal Medical Center, Tokyo University of Agriculture and Technology, Fuchu-shi, Tokyo, Japan

**Keywords:** cilostazol, bradyarrhythmia, canine, pacemaker implantation, sick sinus syndrome

## Abstract

Recently, cilostazol, a phosphodiesterase III inhibitor, has been described as alternative medical treatment for canine bradyarrhythmia in cases for which pacemaker implantation was not indicated or available. In this retrospective study, we investigated the use and efficacy of cilostazol in dogs with bradyarrhythmia in Japan. Dogs that had been brought to the Tokyo University of Agriculture and Technology Animal Medical Center and 23 veterinary hospitals in Japan and been treated with cilostazol initially as the only therapeutic strategy for bradyarrhythmia between January 2010 and August 2021 were included in this study. Survival analyses were performed using Cox proportional hazards analysis, the log-rank test, and the generalized Wilcoxon test to evaluate the efficacy of cilostazol. Fifty-nine privately owned dogs were included in this study. In the survival time analysis, the risk of death was significantly lower and the survival rate was higher in cases in which cilostazol was administered at 10 mg/kg or more per dose. A third-degree atrioventricular block also significantly increased the risk of death and was associated with a lower survival rate. However, in some patients with a third-degree atrioventricular block, there was an increase in the ventricular rate and improvement in clinical symptoms without disappearance or decrease of the atrioventricular block. This study had several important findings that have not previously been reported concerning the use of cilostazol for canine bradyarrhythmia, including the appropriate dose in a clinical setting and the efficacy and prognosis according to the type of bradyarrhythmia.

## Introduction

Bradyarrhythmia is the general term used to describe a slower than cut-off (<60 bpm) (1) and the most common bradyarrhythmias in dogs include sinus bradycardia, sinus arrest, sinoatrial block, sick sinus dysfunction or syndrome (SSS), and atrioventricular (AV) blocks (2–5). In some of the aforesaid bradyarrhythmias, the decreased heart rate may be not adequately counterbalanced by a proportionally increase of stroke volume, leading to a decrease cardiac output that, in turns, may be associated with cerebral ischemia and signs such as collapse, transient loss of consciousness and sudden death (6).

In human medicine, pacemaker implantation (PMI) is the first-line treatment for symptomatic bradyarrhythmia. Similarly, PMI is the best option for dogs and cats with syncopal episodes (2). However, in veterinary medicine, the limited number of institutions capable of performing PMI, the difficulties encountered in obtaining devices, and the tendency for owners to be willing to consent to a procedure under general anesthesia because the animal is elderly or has an underlying disease means that PMI is often not a realistic option. Therefore, drug treatment may be used for some dogs and cats with bradyarrhythmia. The drugs conventionally used to treat bradyarrhythmia in dogs include the parasympathetic nerve blocker atropine sulfate (3), the sympathetic nerve β-receptor agonists isoproterenol (4) and terbutaline (6), and the combined phosphodiesterase blocker and adenosine receptor antagonists aminophylline (5) and theophylline (7). However, these agents may have limited efficacy is some dogs, and in many cases the long-term prognosis remains anyhow poor despite a possible improvement in life quality (6).

Cilostazol, a phosphodiesterase III inhibitor, has recently been reported to be effective in the treatment of bradyarrhythmia in both humans (8–11) and animals [SSS (12, 13)]. Cilostazol is a platelet aggregation inhibitor that is used in humans to prevent recurrence of cerebral infarction or chronic arterial occlusion. One of the well-known side effects of cilostazol is tachycardia, which led to the use of this agent for bradyarrhythmia. In veterinary medicine, although there have been case reports of the effectiveness of cilostazol in dogs (12, 13), there has been no large-scale study of its efficacy or the optimal dose. Thus, although cilostazol is used in dogs, the “standard dose” remains unknown at this time.

The objective of this retrospective study was to investigate the use of cilostazol in Japanese veterinary hospitals and clinics as a medical treatment for bradyarrhythmia in dogs, its efficacy in terms of symptomatic improvement and prognosis, and the optimal dose based on clinical cases.

## Materials and methods

### Study subjects

The study subjects were privately owned dogs treated with cilostazol for bradyarrhythmia showing clinical symptoms at the Tokyo University of Agriculture and Technology Animal Medical Center or one of 23 veterinary hospitals or clinics in Japan between January 2010 and August 2021. All veterinarians responsible for the treatment of these animals were board-certified by the Japanese Society of Veterinary Cardiology. The enrolled dogs had undergone a complete diagnostic work-up (i.e., blood tests like complete blood cell count, biochemistry, and electrolyte measurements) to rule out any other factor leading to a transient bradyarrhythmia that subsequently improves. The timing of the return visit after cilostazol administration was left to the discretion of the practice facility and the veterinarian in charge.

### Study variables

Information were collected on age at time of diagnosis of bradyarrhythmia, breed, sex, presence of diseases and drug administration (e.g., opioids) that can cause bradyarrhythmias (14), and concomitant heart disease. The drugs used before administration of cilostazol and cardiovascular drugs used in combination with cilostazol were also investigated. Furthermore, the type of bradyarrhythmia, improvement or otherwise of clinical symptoms, and electrocardiographic (ECG) changes after use of cilostazol were collected. In principle, ECG recording was performed by Holter ECG for 3 days, but some cases of sinus bradycardia were performed by standard-limb lead ECG (unipolar lead, I, II, and III; unipolar amplification lead: aVR, aVL, and aVF) recording lasting longer than 3 min. Bradyarrhythmia variables included sinus bradycardia [more than 3 successive sinus complexes at a heart rate <60 bpm (15)], sinus arrest [RR intervals >2.0 s (16)], second-degree AV block [a P wave not related to a QRS complex (17)], advanced second-degree AV block [an AV conduction ratio of 3:1 (two unconducted P wave and one conducted P wave for each QRS complex) or higher (18)], third-degree AV block [as a complete AV block; P wave and QRS complex appear independently (18)]. Second-degree AV blocks were separated into Mobitz type I (Wenckebach) and II; the former shows prolongation of PQ intervals before the QRS complex drops out, whereas the latter does not have prolonged PQ intervals. Additional arrhythmogenic entities that have been looked for included neurally mediated syncope, which was suspected when electrocardiographic patterns suggesting a vagal cardioinhibitory effect (e.g., progressive sinus bradycardia until sinus arrest, progressive sinus bradycardia followed by high-grade atrioventricular blocks with a concomitant decrease in sinus rate, atrial fibrillation followed by a progressive decrease in the ventricular response until ventricular arrest) were preceded by trigger situations like coughing, urination, vomiting, defecation or extreme emotion (1); and sick sinus syndrome, which was suspected in the light of sinus bradycardia, sinus arrest, sino-atrial block of various degrees and/or periods of supraventricular arrhythmia followed by prolonged sinus pauses (1).

In this study, syncope was defined as a transient loss of consciousness resulting from inadequate cerebral perfusion [given the underlying arrhythmogenic substrate of our study population (i.e., bradyarrhythmic dogs), the primarily trigger for such a hypoperfusion was considered the ongoing bradyarrhythmia], while the term collapse was used in the case of sudden loss of postural tone, which was not necessarily accompanied by a loss of consciousness (19).

We also investigated the dose of cilostazol used, survival time after starting on cilostazol, the cause of death in animals that died, and, if appropriate, the reason for discontinuation or interruption of cilostazol. The dogs were classified as dead for either arrhythmia-related or -unrelated causes, and we asked the attending veterinarian about abnormal findings that could be the causes of death in the latter.

### Endpoints

The primary endpoint was arrhythmia-related death or PMI because of bradyarrhythmia that was not controlled by cilostazol. With reference to a study of dogs by Borgarelli et al. (20), arrhythmia-related death in this study was defined as death occurring because of progression of clinical signs of bradycardia. Dogs that were euthanized because of refractory bradycardia were scored as cardiac-related deaths. In this study, sudden death was defined as death occurring during sleep or activity such as walking, or within 2 h after the dog showed sudden signs of heart failure (dyspnea). Sudden death was regarded as cardiac-related if no other cause of death was obvious. The cause of death was determined by the veterinarian in charge of the dog. The data cut-off points were death unrelated to bradyarrhythmia in the opinion of the attending veterinarian, discontinuation of cilostazol, and survival while still receiving cilostazol at the end of the study period. The secondary endpoint was death from any cause, including arrhythmia-related death or PMI; the data cut-off points were discontinuation of cilostazol and survival while continuing cilostazol at the end of the study period.

The number of days from the start of treatment with cilostazol until the endpoint or data cut-off point was reached was calculated for each study subject and taken as the survival time.

### Optimal cilostazol dose

The subjects were divided into groups according to the cilostazol dose administered, based on the methodology from a previous experimental study on healthy dogs: group A, 0–5.0 mg/kg; group B, 5.1–7.5 mg/kg; group C, 7.6–9.9 mg/kg; and group D, ≥10.0 mg/kg (21). A stratified analysis of survival time was performed using log-rank (Cochran-Mantel-Haenszel) tests, and Kaplan-Meier survival curves were constructed. The optimal dose was defined as the dose identified by the above analysis as providing the best therapeutic outcome.

### Survival time

The primary and secondary endpoints were initially investigated in univariate Cox proportional hazards models to identify variables that were potentially associated with time to endpoint, and the hazard ratios (HRs) and 95% confidence intervals (CIs) were calculated. The designated variables were age at diagnosis of bradyarrhythmia, sex (male, neutered male, female, or spayed female), concomitant heart disease, presence of third-degree AV block, and whether the dose was equal to or greater than the optimal dose.

We then conducted multivariate Cox proportional hazard analysis incorporating variables that were statistically significant (*p* < 0.1) in univariate analysis using a step-down procedure, with the final model reached when the *p*-value was < 0.1 for all the remaining variables. We calculated the HRs and 95% CIs for the variables that remained in the final model.

We also stratified variables expected to be strongly associated with the therapeutic efficacy of cilostazol and its prognosis based on the univariate and multivariate analyses. We compared survival rates using log-rank (Cochran–Mantel–Haenszel) and generalized Wilcoxon (Gehan–Breslow) tests and produced Kaplan–Meier survival curves.

All statistical analyses were performed using Excel-based statistical software (BellCurve for Excel, SSRI, Tokyo, Japan). A *p*-value < 0.05 was considered statistically significant.

## Results

### Case profiles

The study subjects comprised 59 dogs [of which Miniature Dachshund (*n* = 10), Miniature Schnauzer (*n* = 8), and Shiba Inu (*n* = 8) were the most common three breeds], with a median age of 11 years (range 1–16; Table 1). There were nine males, 10 females, 12 neutered males, and 28 spayed females.

**Table 1:** Composition of dog breeds in this study.

**Breed**	**Number**
Miniature Dachshund	10
Miniature Schnauzers	8
Shiba Inu	8
American Cocker Spaniel	6
Toy Poodle	4
Chihuahua	4
Shih Tzu	3
Pomeranian	3
Cairn Terrier	2
West Highland White Terrier	2
Cavalier King Charles Spaniel	2
Pekingese	2
Tibetan Spaniel	1
Whippet	1
Yorkshire Terrier	1
Miniature Bull Terrier	1
Mix	1
Total number	59

There was elevated blood urea nitrogen in seven dogs [41.9 (34.3–87.2) mg/dL, reference value 9.2–29.2 mg/dL], elevated blood creatinine levels in one dog (1.7 mg/dL, reference value 0.4–1.4 mg/dL), elevated alanine aminotransferase in six dogs [181.5 (137–1,119) IU/L, reference value 17–78 U/L], elevated aspartate aminotransferase in one dog (76 IU/L, reference value 17–44 U/L), and elevated alkaline phosphatase in 15 dogs [531.4 (229–3,500) IU/L; reference value 24–117 U/L]. No electrolyte imbalances were found in any dog. In addition, this study did not include dogs treated with drugs that reduce heart rate, such as opioids, antidepressants, beta blockers, calcium channel blockers, or digitalis (14). Twelve dogs had no disease other than arrhythmia. Thirty-seven dogs had some type of heart disease. Of these, 10 dogs had only heart disease, and 27 dogs had both heart disease and other diseases. Among dogs with non-cardiac diseases, four had hypothyroidism, two had Addison's disease, two had tracheal collapse, two had pulmonary fibrosis, one had chronic rhinitis, and one had inflammatory bowel disease. In addition, there were three dogs with liver tumor and three with spleen tumor (Table 2).

**Table 2:** Diseases of patients other than heart disease.

**Disease**	**Number**
Pancreatitis	6
Chronic renal failure	5
Hypothyroidism	4
Inflammation of urinary bladder	3
Liver tumor	3
Spleen tumor	3
Mammary gland tumor	3
Tracheal collapse	2
Pulmonary fibrosis	2
Intervertebral disk displacement	2
Addison's disease	2
Epilepsy	2
Glaucoma	1
Adrenal tumor	1
Chronic rhinitis	1
Congenital retinal atrophy	1
Inflammatory bowel disease	1
Mast cell tumor of skin	1
Periodontal disease	1
Total^a^	44
No disease other than arrhythmia	23

a In the case of complications, each disease is counted.

In all dogs with non-cardiac disease, treatment for these diseases has been performed for at least 6 months prior to cilostazol administration. In addition, the dosages of drugs for underlying disease did not increase or decrease during the cilostazol administration period. Six dogs were treated with a drug that had a positive chronotropic effect prior to cilostazol administration. Aminophylline was used in four dogs and atropine sulfate was used in one dog, neither of which had any effect on bradycardic arrhythmia or its symptoms. These drugs were discontinued with the start of cilostazol.

### Concomitant heart disease

Of the 37 dogs with heart disease, the most common type was myxomatous mitral valve disease (MMVD) alone (17 dogs, 29%) followed by MMVD with myxomatous tricuspid valve disease (MTVD; 16 dogs, 27%). There were also cases of MTVD alone (two dogs, 3%), MMVD with MTVD and aortic regurgitation (one dog, 2%), and ventricular septal defect (one dog, 2%).

Twenty-one dogs had been taking cardiovascular medications for at least 1 month before cilostazol administration. The remaining 10 dogs were not taking cardiovascular medications. Pimobendan (14 dogs; median 0.25 mg/kg twice a day, range for use 0.2–0.5 mg/kg twice daily), furosemide (eight dogs; median 1 mg/kg twice a day, range for use 0.2–2 mg/kg twice daily), angiotensin converting enzyme inhibitor (seven dogs; median 1 mg/kg twice a day, range for use 0.5–3 mg/kg twice daily), isosorbide dinitrate (three dogs; median 1 mg/kg twice a day, range for use 0.5–2 mg/kg twice daily), and amlodipine (0.1 mg/kg twice a day), sildenafil (1 mg/kg three times a day), and beraprost Na (2 μg/kg twice a day; 1 dog each) were among the drugs used. With a few exceptions, treatment was basically performed according to the American College of Veterinary Internal Medicine consensus guidelines for diagnosis and treatment of each heart disease (31 dogs) (22). Each of these medicines was prescribed in combination in 10 dogs. Control of heart disease was achieved in all dogs, with or without cardiovascular medication, at the start of cilostazol administration. Administration of these cardiovascular drugs had been continued during cilostazol administration.

### Type of bradyarrhythmia

The most common type of arrhythmia was sinus arrest (24 dogs, 41%), followed by third-degree AV block (11 dogs, 19%) and sinus bradycardia (10 dogs, 17%). In addition, SSS mainly expressed in the form of alternation of phases of bradyarrhythmias and supraventricular tachycardia (i.e., the so called “bradycardia-tachycardia” form of SSS; six dogs, 10%), advanced second-degree AV block, and neurally mediated syndrome were recorded (Table 3).

**Table 3:** Diagnosis name of bradyarrhythmia included in this study.

**Diagnosis**	**Number**	**Ratio**
Third-degree atrioventricular block	11	19%
Advanced second-degree atrioventricular block	4	7%
Sinus bradycardia	10	17%
Sinus bradycardia + Mobitz type II second-degree atrioventricular block	1	2%
Sinus arrest	24	41%
Bradycardia-tachycardia syndrome	6	10%
Neurally mediated syncope	2	3%
Mixed sinus rhythm and atrioventricular node rhythm, ventricular rhythm, ventricular tachycardia	1	2%
Total number	59	100%

### Changes in clinical symptoms after administration of cilostazol

All 59 dogs had clinical symptoms believed to have been caused by bradyarrhythmia, such as decreased activity, syncope, decreased appetite, collapse, vomiting, and exercise intolerance. Improvements in clinical symptoms were observed after administration of cilostazol in 52 dogs (88%). The most common improvement was recovery from reduced activity, seen in all 14 dogs with reduced activity. In addition, disappearance (17 of 31 dogs) and decrease in frequency (10 of 31 dogs) of syncope were recognized. The degrees of improvement of other clinical symptoms were also high, including decreased appetite (five of six dogs), collapse (eight of nine dogs), vomiting (three of four dogs), and exercise intolerance (one of two dogs). There was no improvement in clinical symptoms in seven dogs (12%). However, there were no cases of exacerbation of clinical symptoms after administration of cilostazol (Table 4).

**Table 4:** Clinical symptoms before cilostazol administration and improvement after administration.

**Clinical symptoms**		**Improvement**		
	**Number**		**Number**	**Ratio**
Syncope	31	Disappearance	17	55%
		Decreased frequency	10	32%
		Total		87%
Collapse	9		8	89%
Decrease of activity	14		14	100%
Decrease of appetite	6		5	83%
Vomiting	4		3	75%
Exercise intolerance	2		1	50%
		No improvement	7/59	12%
		Exacerbation	0/59	0%

### ECG changes after administration of cilostazol

ECG tests were performed 11 [7–180] days after initiation of cilostazol. ECG changes in arrhythmias were evident after administration of cilostazol in 43 dogs (73%). In patients with sinus arrest, a reduction in the duration of sinus arrest (before administration, 6.1 ± 2.7 s vs. after, 3.5 ± 2.2 s) and an increase in heart rate were observed in 20 of 24 dogs (before administration, 73.7 ± 38.4 bpm vs. after, 109.0 ± 40.6 bpm; average heart rate per day, respectively). In addition, a decrease in the frequency of sinus arrest was also observed in 17 dogs.

In patients with sinus bradycardia, an increase in heart rate was observed in eight of 10 dogs (before administration, 60.8 ± 15.7 bpm vs. after, 96.1 ± 31.4 bpm; average heart rate per day, respectively). Holter ECG could be performed on seven of the 10 dogs, but the remaining 3 dogs had only standard-limb lead ECG.

In patients with third-degree AV block, an increase in the number of ventricular beats was observed in three of 10 dogs (30–120, 12–59, and 56–73 bpm, respectively).

In patients with advanced-second degree AV block, block disappearance was observed in three of four dogs, and the frequency of block occurrence was reduced in one dog.

In addition, shortened duration of sinus arrest in patients with neuromodulatory syncope, and increased heart rate and disappearance of block in patients with sinus bradycardia + Mobitz type II second-degree AV block were observed.

No change was evident in 15 dogs (25%). One dog (2%) did not undergo ECG after administration of cilostazol. There were no cases of exacerbation of ECG findings (Table 5).

**Table 5:** Changes in ECG findings after administration of cilostazol.

**Diagnosis**	**ECG changes**	**Number**	**Ratio**
Improvement		43/59	73%
Third-degree AVB			
	Increased ventricular beat rate	3/11	
Advanced second-degree AVB			
	Disappearance of AVB	3/4	
	Decrease in the frequency of occurrence of AVB	1/3	
Sinus bradycardia			
	Increased heart rate	8/10	
Sinus arrest			
	Shortening the duration of sinus arrest + Decrease in the frequency of occurrence of sinus arrest + Increased heart rate	17/24	
	Shortening the duration of sinus arrest + Increased heart rate	3/24	
SSS type III (bradycardia-tachycardia syndrome)			
	Shortening the duration of sinus arrest	3/6	
Neurally mediated syncope			
	Shortening the duration of sinus arrest	2/2	
Sinus bradycardia + Mobitz type II second- degree AVB		1/1	
	Increased heart rate + disappearance of AVB		
No improvement		15/59	25%
Exacerbation		0/59	0%
Not clear		1/59	2%
Total number		59	100%

AVB, Atrioventricular block.

### Reasons for discontinuation

Forty-one dogs continued treatment with cilostazol until death (28 dogs, 47%) or until August 2021 (13 dogs, 22%). Cilostazol was discontinued in 12 cases (20%) for reasons that included PMI (five dogs, 8%, 14–1,178 days), improvement in arrhythmia (two dogs, 3%, 90 and 609 days), and lack of effect (two dogs, 3%, 14 and 69 days). Cilostazol was also discontinued immediately by the owner because of development of clinical symptoms considered to be adverse effects in two dogs (3%). In 1 of these cases, the dog vomited 14 days after administration of cilostazol, and in the other the dog developed atrial flutter 180 days after starting treatment with this agent. The latter was suspected to be an adverse effect caused by cilostazol because no cardiac disease with severe atrial enlargement was identified and the patient had not received any other medications. However, a microscopic atrial myopathy could not be completely excluded and could have represented a concomitant arrhythmogenic substrate. Moreover, it should be also considered that atrial flutter was the result of other confounding factor, such as a fluctuation of the vagal stimulation of the sinus node (i.e., “functional atrial flutter”) not necessarily related to the administration of cilostazol (23, 24). All dogs who discontinued cilostazol were not re-administered.

### Dosage and administration of cilostazol

The dosages and timing of administration of cilostazol were selected based on past case reports (12, 13), basic research (21), and the experience of the attending veterinarian. The most frequently used dose of cilostazol was 10.0–10.9 mg/kg twice daily (20.0–21.8 mg/kg/day). The next most frequently used dose was 5.0–5.9 mg/kg twice daily (10.0–11.8 mg/kg/day). The lowest daily dose was 3.5 mg/kg/day and the highest was 37.5 mg/kg/day. The frequency of number of doses was once daily in two dogs, twice daily in 52 dogs, and three times daily in five dogs. The median duration of medication in this study was 288 days (range 2–2,453 days).

### Endpoints reached

By the end of the study period, 17 of the 59 study subjects reached the primary endpoint (12 arrhythmia-related deaths, five cases of PMI). The secondary endpoint was reached by 33 dogs (12 arrhythmia-related death, five cases of PMI, 16 deaths unrelated to arrhythmia). The causes of death unrelated to arrhythmia included debilitation associated with aging and senility (six dogs), renal failure (four dogs), left-sided congestive heart failure due to MMVD and lung edema (two dogs), and pancreatitis, lung tumor, aspiration pneumonia, and unknown causes (one dog for each).

### Optimal cilostazol dose

There were 13 dogs in group A, 8 in group B, 12 in group C, and 26 in group D. A stratified analysis of survival time showed that the survival rate was highest in group D (≥10.0 mg/kg); this dose was considered to have a good therapeutic outcome. The optimal dose of cilostazol was determined to be 10.0 mg/kg.

### Survival time

Univariate Cox hazard analysis showed that third-degree AV block significantly increased the risk of reaching the primary endpoint (HR 3.74, 95% CI 1.42–9.83, *p* < 0.01) and that a cilostazol dose of ≥10 mg/kg significantly reduced the risk of reaching the secondary endpoint (HR 0.38, 95% CI 0.18–0.80, *p* = 0.01; Table 6). Advancing age also tended to increase the risk of reaching the secondary endpoint (HR 1.09, 95% CI 0.99–1.20, *p* = 0.07; Table 6).

**Table 6:** Hazard ratio for reaching primary and secondary endpoints by univariate Cox proportional hazards analysis.

	**The primary endpoint**			**The secondary endpoint**		
**Variable**	**HR**	**95% CI**	***p*-value**	**HR**	**95% CI**	***p*-value**
Age	0.94	0.84–1.06	0.31	1.09	0.99–1.20	0.07
Sex (females or spayed females)	1.07	0.39–2.90	0.90	0.81	0.40–1.66	0.57
Concomitant heart disease	0.70	0.27–1.86	0.48	0.84	0.41–1.72	0.63
Third-degree atrioventricular block	3.74	1.42–9.83	<0.01	1.82	0.84–3.97	0.13
Cilostazol dose≥10 mg/kg	0.40	0.14–1.14	0.09	0.38	0.18–0.80	0.01

HR, Hazard rate; CI, Confidence Interval.

The primary endpoint could not be investigated by multivariate Cox hazard analysis because of the small sample size. In multivariate analysis for the secondary endpoint, the final model incorporated the three variables of age, third-degree AV block, and a cilostazol dose ≥10 mg/kg. Advancing age and third-degree AV block significantly increased the risk of reaching the secondary endpoint (age, HR 1.14, 95% CI 1.03–1.27, *p* = 0.01; third-degree AV block, HR 2.98, 95% CI 1.30–6.88, *p* = 0.01; Table 7). A cilostazol dose ≥10 mg/kg significantly decreased the risk of reaching the secondary endpoint (HR 0.34, 95% CI 0.15–0.74, *p* < 0.01; Table 7).

**Table 7:** Hazard ratio for reaching secondary endpoint by multivariate Cox proportional hazards analysis.

**Variable**	**HR**	**95% CI**	***p*-value**
Age	1.14	1.03–1.27	0.01
Third-degree atrioventricular block	2.98	1.30–6.88	0.01
Cilostazol dose ≥ 10 mg/kg	0.34	0.15–0.74	<0.01

HR, Hazard rate; CI, Confidence Interval.

**Figure 1:**
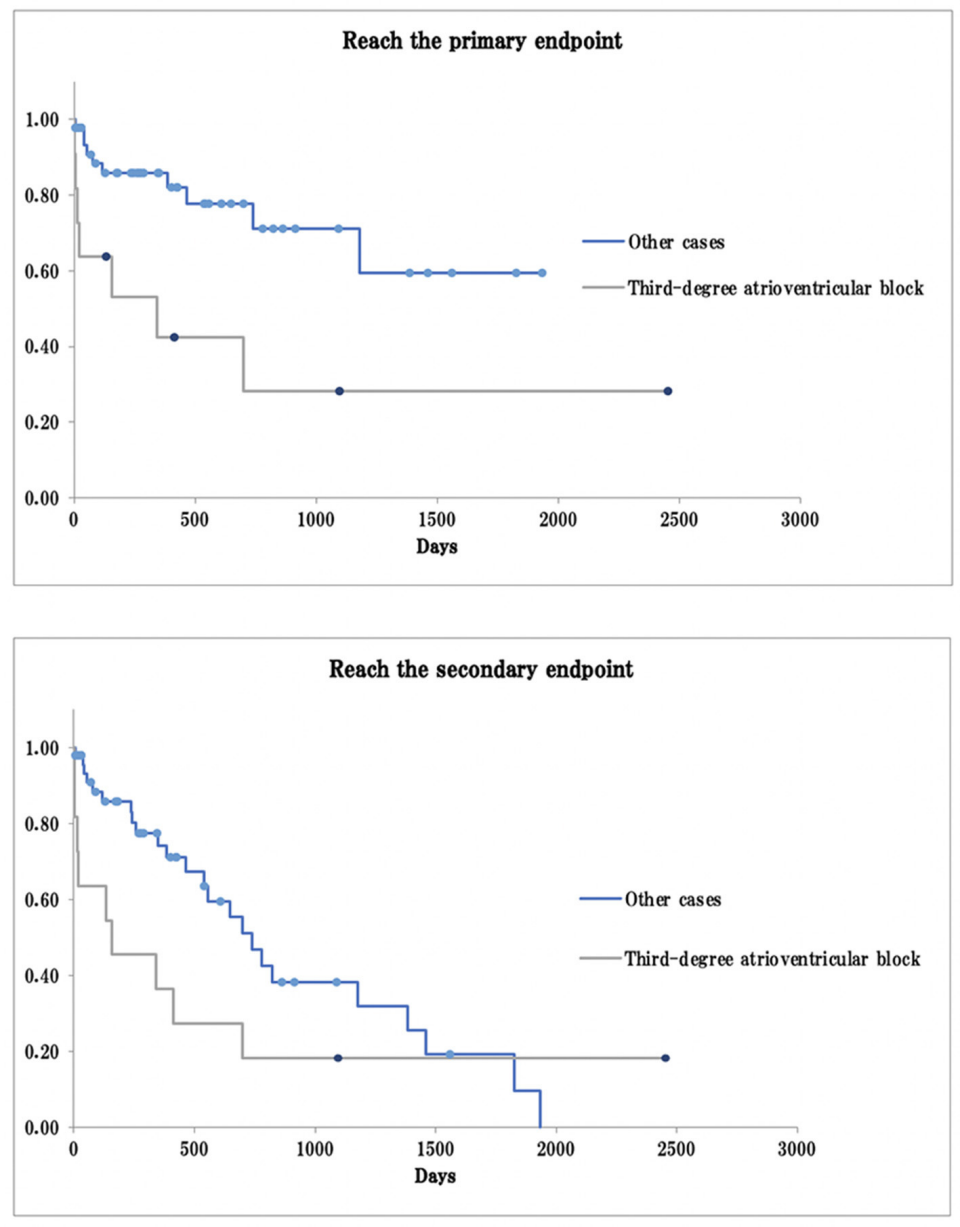
Survival curve in third-degree atrioventricular block and other cases. The primary endpoint: arrhythmia-related death or PMI as a result of bradyarrhythmia that was not controlled by cilostazol; The secondary endpoint: death from any cause, including arrhythmia-related death or PMI; Dot: the data cut-off points.

**Figure 2:**
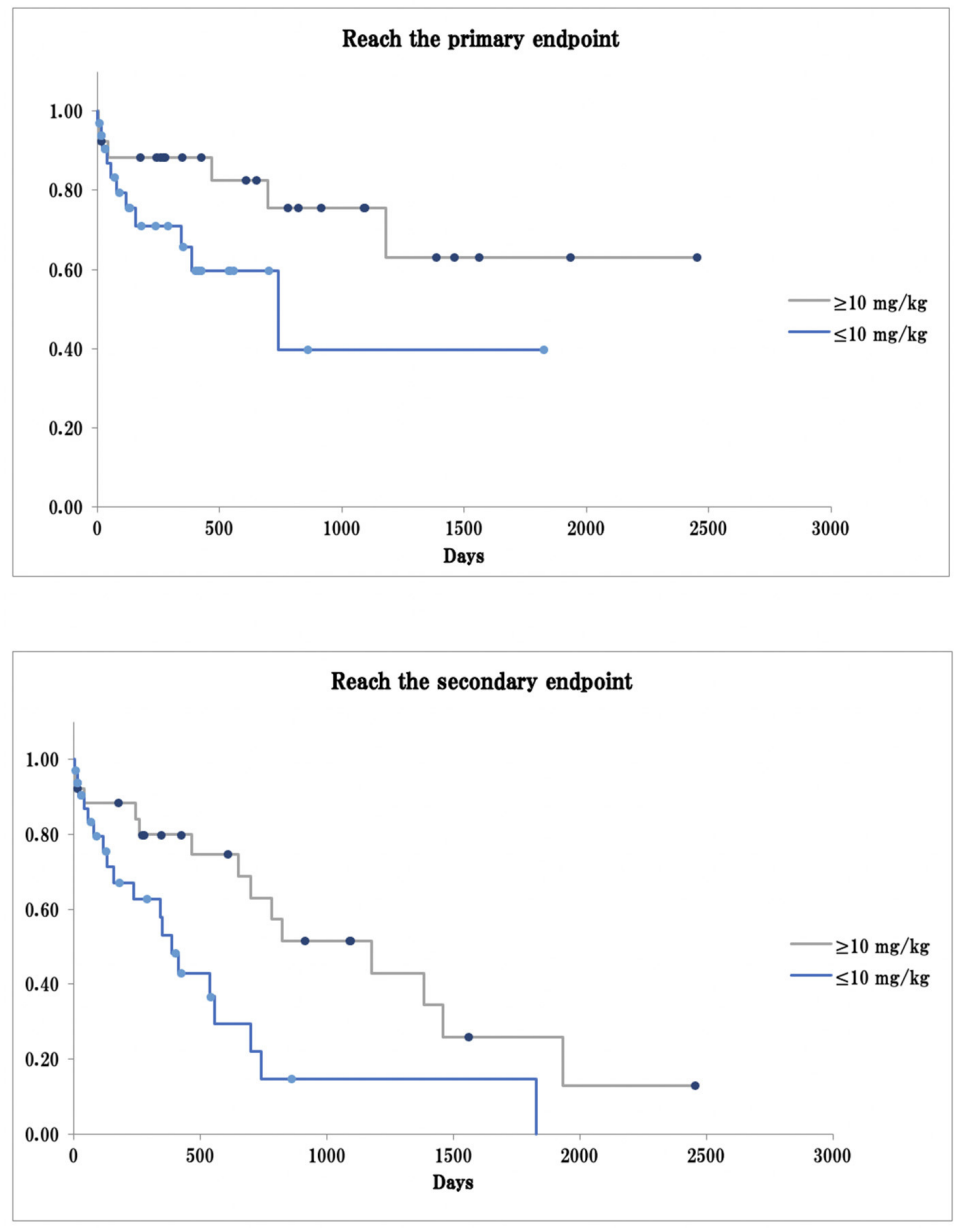
Survival curve in cilostazol dose ≥10 mg/kg or ≤ 10 mg/kg. The primary endpoint: arrhythmia-related death or PMI as a result of bradyarrhythmia that was not controlled by cilostazol; The secondary endpoint: death from any cause, including arrhythmia-related death or PMI; Dot: the data cut-off points.

Univariate and multivariate Cox proportional hazards analyses showed that dogs with third-degree AV block took significantly less time to reach the primary and secondary endpoints than dogs with other types of bradyarrhythmia (primary endpoint, log-rank test *p* < 0.01, generalized Wilcoxon test *p* < 0.01; secondary endpoint: log-rank test, *p* = 0.13, generalized Wilcoxon test *p* < 0.01; Figure 1). Dogs receiving a cilostazol dose ≥10 mg/kg took more time to reach the primary endpoint than those receiving a dose ≤ 10 mg/kg, although the difference was not statistically significant (log-rank test *p* = 0.08, generalized Wilcoxon test *p* = 0.14). These dogs also took significantly longer to reach the secondary endpoint (log-rank test *p* < 0.01, generalized Wilcoxon test *p* = 0.03; Figure 2).

## Discussion

This study had several important findings that have not previously been reported concerning the use of cilostazol in Japan for canine bradyarrhythmia, the appropriate dose in a clinical setting, and the efficacy and prognosis according to type of bradyarrhythmia.

Sick sinus syndrome (SSS) is more common in Miniature Schnauzers, Dachshunds, Cocker Spaniels, West Highland White Terriers, Pugs, and in middle-aged and older females, with potential involvement of genetic factors (25–27). In this study, sinus bradycardia, sinus arrest, and bradycardia-tachycardia syndrome, which are manifestations of SSS, were present in 41 of 59 dogs. Three breeds accounted for most of these cases: Miniature Schnauzers (eight dogs, 20%), Miniature Dachshunds (seven dogs, 17%), and American Cocker Spaniels (five dogs, 12%). By sex, 10 (24%) of these 41 dogs were males or neutered males and 31 (76%) were females or spayed females, with females clearly accounting for the majority. The profile of these dogs with SSS was thus consistent with that reported previously (25–27).

On the other hand, there are reports suggesting that the incidence of AV block in dogs is higher in some breeds, including Dachshunds and Cocker Spaniels, which develop idiopathic fibrosis and age-related prolongation of the PR interval (28). Similarly, dog breeds such as Doberman Pinschers and Pugs, which are prone to the loss of the bundle of His, have also been reported to have a higher incidence (28). In the present study, four of 16 (25%) dogs with AV block were Shiba Inus and three dogs (19%) were Miniature Dachshunds described in a previous report (25–27). The large number of Shiba Inus in this study likely reflects the popularity of this breed in Japan (29). There was no sex bias, with eight dogs each (50%) being male/neutered male or female/spayed female. As mentioned above, although certain breeds of dog are reportedly more susceptible to AV block, a wide variety of factors have been implicated in its etiology, including drugs, electrolyte imbalance, and pathological changes in the AV node (30–33), and it has been suggested that advanced second-degree AV block and third-degree AV block are degenerative disorders that become more likely with advancing age (18). Therefore, genetic factors may have less of an effect on AV block compared with SSS. There may also be less sex bias.

The most common type of bradyarrhythmia was sinus arrest (24 dogs, 41%) and the next most common was AV block (11 dogs, 19%). Uchino et al. analyzed Holter ECG recordings from dogs with clinical symptoms of falls, exercise intolerance, and syncope, and calculated the incidence of each type of arrhythmia (34). They found that sinus arrest was the most common type, accounting for 22.0% of arrhythmias, and that third-degree AV block accounted for 4.7%. The difference in incidence between that study and the present study may reflect the fact that Uchino et al. also included tachyarrhythmias whereas our study was limited to bradyarrhythmias.

Bradycardic arrhythmias in dogs are due to electrolyte abnormalities, drug administration with negative diachronic effects, certain hormonal disorders that affect autonomic function, chronic respiratory disorders, and chronic gastrointestinal disorders that cause increased vagal tone. It can be caused by a disease or a reversible condition such as a peritoneal tumor involving the vagal trunk. Treatment for them may result in the disappearance or improvement of bradyarrhythmias. The dogs in this study had no history of electrolyte imbalances or negative chronotropic medications. On the other hand, dogs with hypothyroidism, Addison's disease, pulmonary fibrosis, tracheal collapse, inflammatory bowel disease, liver tumor, and spleen tumor were included. All these dogs had been treated according to veterinary textbooks 6 months before cilostazol administration and did not undergo a change in treatment for the target disease during cilostazol administration, at least until the ECG. Therefore, it was determined that the presence of these diseases had little effect on the outcome of cilostazol administration.

Myxomatous mitral valve disease (MMVD) was the most common concomitant heart disease in our study subjects; the most common was MMVD alone (17 dogs, 29%) followed by MMVD with MTVD (16 dogs, 27%). All dogs with MMVD in this study had stage B1 or B2 disease according to the American College of Veterinary Internal Medicine classification (35) at the time of diagnosis of bradyarrhythmia, and thus had no present or past signs of heart failure. None of the other concomitant heart diseases seen in the dogs in this study were considered particularly serious at the time bradyarrhythmia was diagnosed. Age-related pathologic changes in the mitral and tricuspid valves, known as myxomatous degeneration, are well-known in dogs. Many dogs with MTVD also have MMVD. A large proportion of our study subjects were middle-aged or older, which is the typical age for onset of myxomatous degeneration (35), and were receiving treatment with drugs other than cilostazol. However, the HR for concomitant heart disease was <1.0. The above findings suggest that the heart disease seen in the dogs in this study was either mild or well-controlled by pimobendan and had a negligible effect on the efficacy of cilostazol *per se*.

The efficacy of cilostazol was evident in the fact that the duration and frequency of sinus arrest decreased in 20 (83%) of the 24 dogs with sinus arrest. Cilostazol has been shown to exert a positive chronotropic effect on excised sinus nodes (36), suggesting that it may act directly on the sinus node. Possible mechanisms for this action include promotion of the automaticity of sinus node cells by activation of intracellular signal transduction *via* cAMP and improvement of sinus node function by increasing coronary artery perfusion in experimental dogs (37, 38). Therefore, cilostazol may be an effective treatment for sinus bradycardia, sinus arrest, and SSS, all of which are arrhythmias caused by sinus node dysfunction. We have previously demonstrated that cilostazol significantly reduces the PQ interval in dogs, which indicates that this agent influences AV conduction time but not the QT interval (21). Therefore, cilostazol may be effective in patients with maintained AV conduction or in patients with abnormal but not yet completely disrupted AV conduction. In our study, we also found that all four dogs with advanced second-degree AV block had a reduced frequency of block occurrences per day or the block itself disappeared.

An increase in the ventricular response rate of 10–100 bpm was observed in seven of 11 dogs (64%) with third-degree AV block, although the block did not disappear on ECG in any of these 11 cases. Similar results have been reported previously in canine (12) and human (9) patients with third-degree AV block, with increased escape rhythms observed even though the AV block itself had not disappeared. Although the mechanism by which cilostazol increases the ventricular response in patients with third-degree AV block remains unknown, its actions may involve dilation of the coronary vessels (37) and increasing cAMP (39). Improvement in clinical symptoms was evident in eight of 11 dogs (73%) with third-degree AV block after administration of cilostazol. This finding suggests that it may be worth considering use of cilostazol in dogs with third-degree AV block when PMI is not an available option (e.g., due to owners' economic restraints, lack of specialized medical team and/or instrumentation), with the goal of improving quality of life at least to some extent.

However, in this study, the survival rate of dogs with third-degree AV block was significantly lower than that of dogs with other types of arrhythmias. Moreover, these dogs were at high risk of reaching the primary endpoint (nine of 11 dogs, 82%). This may be because our study included dogs with third-degree AV block in which an increased ventricular response either did not occur after administration of cilostazol or an increase did occur but was insufficient to provide the necessary systemic blood supply.

In univariate Cox proportional hazard analysis, advancing age tended to increase the risk of reaching the secondary endpoint but not that of reaching the primary endpoint. Causes of death other than arrhythmia in patients that reached the secondary endpoint in this study included debilitation and renal failure, which are common in elderly dogs. Patients that died from a cause other than arrhythmia accounted for only around half of the dogs that reached the secondary endpoint (16 dogs, 48%). This finding indicates that cilostazol provided long-term control in most cases, with dogs continuing to survive into old age without exacerbation of arrhythmia.

In previous experiments in dogs (21) and in clinical reports (12, 13, 40, 41) the dose of cilostazol used was 5–10 mg/kg twice daily. However, there has been no relevant prospective or retrospective clinical study with a large sample. Therefore, in this study, we conducted an exploratory analysis of the doses used in advance of an overall evaluation by univariate and multivariate analyses. The results of this exploratory analysis showed that the survival rate was highest at a dose of ≥10 mg/kg, which suggests that this dose may achieve long-term survival in dogs with bradyarrhythmia. To confirm this effect, we conducted a Cox proportional hazards analysis incorporating dose ≥10 mg/kg as a variable. We also compared the survival curves for patients receiving doses ≥10 mg/kg and doses <10 mg/kg and found that treatment with a dose ≥10 mg/kg increased the survival rate. Komiya et al. started dogs with Rubenstein type II SSS on a cilostazol dose of 5 mg/kg twice daily and found that this dose produced little change in the ECG; however, when it was increased to 9.3 mg/kg, the duration and frequency of sinus arrest decreased (12). This suggests that the conventional starting dose of 5 mg/kg may not be effective in many cases, and that a dose ≥10 mg/kg is probably more appropriate. The reason for the inconsistency between our study findings and the recommended dose may stem from the fact that the dose of ≥10 mg/kg was derived from previous studies conducted in clinically healthy Beagles (21). However, in everyday clinical practice, patients with bradyarrhythmia requiring treatment are likely to have organic or functional cardiac changes and are often older.

In this study, cilostazol was administered twice daily in most cases (52 of 59 dogs, 88%), with five dogs (8%) receiving three doses a day (9.5 mg/kg in 1 case and ≥10 mg/kg in the other 4). Therefore, the total dose was high. All dogs had originally been treated twice daily, but the dosage had been increased to three times daily because of lack of improvement in symptoms. In three of these cases, symptomatic improvement was evident after the dose had been increased, and treatment with the same dose of cilostazol was continued thereafter. Cilostazol was ineffective in the remaining two dogs, both of which progressed to PMI. Akiyama et al. reported that the half-life of cilostazol in dogs is 1.6 h (42). Fukushima et al. also reported in a study using healthy dogs that a significant increase in heart rate was maintained for 11 h after administration of cilostazol (21). These findings indicate that there may be a period during which the efficacy of cilostazol is not fully maintained when dosing is twice daily. In line with the results of previous studies, our present findings suggest that, in general, if patients with bradyarrhythmia do not respond sufficiently to twice-daily treatment with cilostazol, they may start to respond if the regimen is changed to three times daily because of the half-life of cilostazol (42) and duration of increased heart rate in dogs (21). However, a toxicity study in healthy dogs showed that cilostazol 30 mg/kg/day administered orally for 13 weeks resulted in appearance of cardiac lesions, including coronary arteritis, intracardiac hemorrhage, and hemosiderin deposits and fibrosis in the left ventricle (43), and some cases were administered for longer than 13 weeks in this study, which could have resulted in potential failure. Furthermore, in the present study, we found that a dose of ≥10 mg/kg was desirable, and that dosing three times daily may be more effective than dosing twice daily. However, a dose of resulted in potential failure. Furthermore, in the present study, we found that a dose of ≥10 mg/kg was desirable, and that adverse drug reactions. Therefore, close monitoring is required when administering cilostazol at a dose of ≥10 mg/kg three times daily.

In human medicine, cilostazol has been reported to cause sinus tachycardia (11) and rapid ventricular tachycardia (44) when used in patients with bradyarrhythmias. There were no cases of tachyarrhythmia in this study, except for one dog that developed atrial flutter. At least two cases of atrial flutter have been reported to the Pharmaceuticals and Medical Devices Agency, an independent administrative agency in Japan. However, the causal relationship between cilostazol and atrial flutter is unclear because these patients were receiving multiple drugs. Further follow-up is needed, as this may have been influenced by the duration and dose of administration.

This study has several limitations. The first is the small sample size. We had originally intended to investigate the primary endpoint using a multivariate Cox proportional hazards model. However, the number of cases in which events occurred was too small, so this statistical method had to be abandoned. Many aspects of the use of cilostazol to treat bradyarrhythmia remain unclear, and further studies with larger sample sizes are required. The second limitation is that the judgment as to whether the cause of an individual death was related or unrelated to arrhythmia was made by the attending veterinarian. Causes of death not due to arrhythmia included renal and heart failure. However, it is difficult to exclude the possibility that these deaths may also have been caused by bradyarrhythmia. Third, for many cases in this study, Holter ECG was used to determine the degree of improvement, including heart rate and sinus arrest time before and after cilostazol administration. However, in some cases, it was done by ECG using a standard limb lead. Therefore, it should be noted that the standard limb lead ECG is a momentary test, meaning that the amount of data obtained from the patient is small and the analytical effect is inferior to that of Holter ECG. Fourth, some patients had heart disease and/or non-heart disease in addition to arrhythmias. We tried to eliminate as many factors as possible. However, it cannot be completely ruled out that these diseases may have limited or promoted the effects of cilostazol. Finally, changes in clinical symptoms were based on owners used to determine the degree of improvement, including heart rate and sinus arrest time before and after cilostazol administration. However, in some cases, mated or underestimated.

In this study, we confirmed a clinically beneficial effect of cilostazol in dogs with bradyarrhythmia. Further multifaceted studies are required in dogs that have undergone PMI in terms of factors such as differences in survival time to ascertain the value of administration of this agent in greater detail.

## Data availability statement

The original contributions presented in the study are included in the article/supplementary material, further inquiries can be directed to the corresponding author.

## Ethics statement

Ethical review and approval was not required for the animal study because it is a retrospective study. Veterinarians gave oral informed consent to the use of cilostazol as a therapeutic agent for bradyarrhythmia to the owner, and it was used when consent was obtained. These veterinarians also obtained written consent from the dog owners for permission to use patient data for this study.

## Author contributions

TO and RF: conception and design. AY, SM, HH, and DH: clinical consultation and acquisition of data. TO and YM: data and statistical analysis. YM: drafting the first manuscript. RF: editing and revising manuscript. All authors contributed to the article and approved the submitted version.

## Funding

This study was supported by the Japan Society for the Promotion of Science KAKENHI (Grant No. 26450425).

## Conflict of interest

The authors declare that the research was conducted in the absence of any commercial or financial relationships that could be construed as a potential conflict of interest.

## Publisher's note

All claims expressed in this article are solely those of the authors and do not necessarily represent those of their affiliated organizations, or those of the publisher, the editors and the reviewers. Any product that may be evaluated in this article, or claim that may be made by its manufacturer, is not guaranteed or endorsed by the publisher.

## References

[1] Santilli R Moise NS Pariaut R Perego M. Electrocardiography of the Dog and Cat: Diagnosis of Arrhythmias. 2nd ed. (Italian edition). Milano: Edra Spa (2018). p. 468-501.

[2] Kobayashi M Hoshi K Hirao H Shimizu M Shimamura S Akiyama M. Implantation of permanent transvenous endocardial pacemaker in a dog with atrioventricular block. J Vet Med Sci. (2003) 65:1131–34. 10.1292/jvms.65.1131 14600355

[3] Machida N Yamaga Y Kagota K. Three cases of sinus bradycardia in dogs. J Jpn Vet Med Assoc. (1990) 43:447–50. 10.12935/jvma1951.43.447

[4] Shibasaki A Katamoto H Nomura K. A case of sick sinus syndrome in german shepherd dog. J Anim Clin Med. (2001) 11:143–6.

[5] Alexio ASC Alfonso A Kichise BK Neto FT Girotto CH Garzesi AM. Pacemaker implant in a dog with sick sinus syndrome. Acta Scientiae Veterinariae. (2017) 45:6. 10.22456/1679-9216.85851

[6] Ward JL DeFrancesco TC Tou SP Atkins CE Griffith EH Keene BW. Outcome and survival in canine sick sinus syndrome and sinus node dysfunction: 93 cases (2002-2014). J Vet Cardiol. (2016) 18:199–212. 10.1016/j.jvc.2016.04.004 27286907

[7] Billen F Israel NV. Syncope secondary to transient atrioventricular block in a German shepherd dog with dilated cardiomyopathy and atrial fibrillation. J Vet Cardiol. (2006) 8:63–8. 10.1016/j.jvc.2005.12.003 19083338

[8] Atarashi H Endoh Y Saitoh H Kishida H Hayakawa H. Chronotropic effects of cilostazol, a new antithrombotic agent, in patients with bradyarrhythmias. J Cardiovasc Pharmacol. (1998) 31:534–9. 10.1097/00005344-199804000-00010 9554801

[9] Kodama TK Kurata A Ohshima K Yamamoto K Uemura S Watanabe S. Effect of cilostazol on the ventricular escape rate and neurohumoral factors in patients with third-degree atrioventricular block. Chest. (2003) 123:1161–9. 10.1378/chest.123.4.1161 12684307

[10] Moriya I Takahashi T Nomura Y Kawaura K Kusaka K Yamakawa J. Chronotropic effect of the antithrombotic agent cilostazol in a patient with sick sinus syndrome and syncope. J Int Med Res. (2004) 32:549–51. 10.1177/147323000403200513 15458288

[11] Nimura A Sato N Sakuragi H Koyama S Maruyama J Talib AK. Recovery of advanced atrioventricular block by cilostazol. Intern Med. (2011) 50:1957–61. 10.2169/internalmedicine.50.5228 21921376

[12] Komiya M Sasaki N Tanabe T Ohmori T Fukushima R. A canine case of sick sinus syndrome (Rubenstein-II) successfully treated with cilostazol: findings on monitoring with holter electrocardiography. Adv Anim Cardiol. (2013) 46:43-51. 10.11276/jsvc.46.43

[13] Kanno N Suzuki T. Long term effects of cilostazol in a dog with sick sinus syndrome. J Vet Med Sci. (2017) 79:1031–4. 10.1292/jvms.17-0018 28458273 PMC5487778

[14] Nelson RW Couto CG. Small Animal Internal Medicine. 6th ed. Amsterdam: Elsevier (2019). p. 77–99.

[15] Romito G Gublielmini C Poser H Toaldo MB. Lorenz plot analysis in dogs with sinus rhythm and tachyarrhythmias. Animals. (2021) 11:1645. 10.3390/ani11061645 34206036 PMC8228210

[16] Hall LW Dunn JK Delaney M Shapiro LM. Ambulatory electrocardiography in dogs. Vet Rec. (1991) 129:213–6. 10.1136/vr.129.10.213 1949516

[17] Kittleson MD. Diagnosis and treatment of arrhythmias (dysrhythmias). In: Kittleson MD Kienle RD editors, Small Animal Cardiovascular Medicine, St. Louis, MO: Mosby (1998). p. 449–94.

[18] Schrope DP Kelch WJ. Signalment, clinical signs, and prognostic indicators associated with high-grade second- or third-degree atrioventricular block in dogs: 124 cases (January 1, 1997-December 31, 1997). J Am Vet Med Assoc. (2006) 228:1710–7. 10.2460/javma.228.11.1710 16740072

[19] Martin M. Syncope. In: Ettinger SJ Feldman EC Cote E editors. Textbook of Veterinary Internal Medicine. 8th ed. St. Louis, MO: Elsevier (2017). p. 123–6.

[20] Borgarelli M Savarino P Crosara S Santilli RA Chiavegato D Poggi M. Survival characteristics and prognostic variables of dogs with mitral regurgitation attributable to myxomatous valve disease. J Vet Intern Med. (2008) 22:120–8. 10.1111/j.1939-1676.2007.0008.x 18289298

[21] Fukushima R Kawaguchi T Yamada S Yoshimura A Hirao D Ohmori T. Effects of cilostazol on the heart rate in healthy dogs. J Vet Med Sci. (2018) 80:1707–15. 10.1292/jvms.18-0240 30249936 PMC6261822

[22] Reinero C Visser LC Kellihan HB Masseau I Rozanski E Clercx C. ACVIM consensus statement guidelines for the diagnosis, classification, treatment, and monitoring of pulmonary hypertension in dogs. J Vet Intern Med. (2020) 34:549–73. 10.1111/jvim.15725 32065428 PMC7097566

[23] Romito G Summerfield N Toaldo MB. Presumptive vagally-mediated atrial flutter in a dog. J Vet Cardiol. (2022) 39:46–50. 10.1016/j.jvc.2021.12.006 34973471

[24] Romito G Summerfield N Toaldo MB. Corrigendum to ‘Presumptive vagally-mediated atrial flutter in a dog' [J Vet Cardiol (2022) 39, 46-50]. J Vet Cardiol. (2022) 42:14. 10.1016/j.jvc.2022.05.004 35662024

[25] Rishniw M Thomas WP. Current Veterinary Therapy XIII. Kirk RW editor, Philadelphia, PA: WB Saunders (2000). p. 719–25.

[26] Edwards NJ. Bolton's Handbook of Canine and Feline Electrocardiography. 2nd ed. Philadelphia, PA: WB Saunders (1987). p. 66–151.

[27] Ware AW. Cardiovascular Disease in Small Animal Medicine. 3rd ed. London: Manson Pub/The Veterinary Press (2011). 10.1201/b15177

[28] Tilley LP. Essentials of Canine and Feline Electrography: Interpretation and Treatment. 3rd ed. Philadelphia, PA: Lea & Febiger (1992). p. 127–207.

[29] Uddin MM Arata S Takeuchi Y Chang HS Mizukami K Yabuki A. Molecular epidemiology of canine GM_1_ gangliosidosis in the Shiba Inu breed in Japan: relationship between regional prevalence and carrier frequency. BMC Vet Res. (2013) 9:132. 10.1186/1746-6148-9-132 23819787 PMC3701567

[30] Toaldo MB Romito G Cipone M Diana A Tursi M. Electrocardiographic, echocardiographic, and left atrial strain imaging features of a dog with atrial flutter and third-degree atrioventricular block. J Vet Cardiol. (2017) 19:462–8. 10.1016/j.jvc.2017.08.004 28943125

[31] Romito G Diana A Rigillo A Morini M Cipone M. Unusual presentation of aortic valve infective endocarditis in a dog: aorto-cavitary fistula, tricuspid valve endocarditis, and third-degree atrioventricular block. Animals. (2021) 11:690. 10.3390/ani11030690 33806631 PMC7998688

[32] Kaneshige T Machida N Nakao S Doiguchi O Katsuda S Yamane Y. Complete atrioventricular block associated with lymphocytic myocarditis of the atrioventricular node in two young adult dogs. J Comp Pathol. (2007) 137:146–50. 10.1016/j.jcpa.2007.05.006 17673248

[33] Kaneshige T Machida N Yamamoto S Nakao S Yamane Y. A histological study of the cardiac conduction system in canine cases of mitral valve endocardiosis with complete atrioventricular block. J Comp Pathol. (2007) 136:120–6. 10.1016/j.jcpa.2007.01.001 17362978

[34] Uchino T Kurita T Yamaguchi M. The occurrence of serious ventricular arrhythmia in dogs and cats. J Arrhythm. (2007) 27:71–7. 10.5105/jse.27.Suppl1_71

[35] Keene WB Atkins CE Bonagura JD Fox PR Haggstrom J Fuentes VL. ACVIM consensus guidelines for the diagnosis and treatment of myxomatous mitral valve disease in dogs. J Vet Intern Med. (2019) 33:1127–40. 10.1111/jvim.15488 30974015 PMC6524084

[36] Saito K Ohno N Ono T Nomura T Endou Y Arata H. Usefulness of cilostazol for sinus node syndrome - about cases of manifestation of sinus node due to discontinuation of administration -. Jpn Pharmacol Ther. (1995)23:977–82.

[37] Shintani S Watanabe K Kawamura K Mori T Tani T Toba Y. General pharmacological properties of cilostazol, a new antithrombotic drug. Part II: effect on the peripheral organs. Arzneimittelforschung. (1985) 35:1163–72. 3000392

[38] Yamashita S Miyagawa K Inagaki T Dohi Y. Cilostazol increased heart rate with improvement of activity of daily living in an elderly patient with sick sinus syndrome. Nihon Ronen Igakkai Zasshi. (1995) 36:561–4. 10.3143/geriatrics.36.561 10554564

[39] Okuda Y Kimura Y Yamashita K. Cilostazol. Cardiovasc Drug Rev. (1993) 11:451–65. 10.1111/j.1527-3466.1993.tb00200.x

[40] Fukushima R Araie T Suzuki S Matsumoto H Morita S Aytemiz D. A case of situational syncope due to cough in a dog. J Anim Clin Med. (2017) 26:29–33. 10.11252/dobutsurinshoigaku.26.29

[41] Fukushima R Ohmori T Goya S Nakada M Chantawong P Kawaguchi T. Two dogs with improved renal function after administration of cilostazol. Adv Anim Cardiol. (2017) 50:15–9. 10.11276/jsvc.50.15

[42] Akiyama H Kudo S Shimizu T. The absorption, distribution and excretion of a new antithrombotic and vasodilating agent, cilostazol, in rat, rabbit, dog and man. Arzneimittelforschung. (1985) 35:1133–40.4074424

[43] Nagano K Kitaura K Ohkawa T Hyabuchi T Shintani S. Toxicological studies on cilostazol (V). 13-Week subacute toxicity in dogs. Iyakuhin Kenkyu. (1985) 16:1268–84.

[44] Gamssari F Mahmood H Ho JS Villareal RP Liu B Rasekh A. Rapid ventricular tachycardias associated with cilostazol use. Tex Heart Inst J. (2002) 29:140–2. Retrieved from: https:/meridian.allenpress.com/thij. 12075874 PMC116744

